# Découverte d’un cas inhabituel de méga cholécystite aigue associé à une dilatation kystique de la voie biliaire principale

**DOI:** 10.11604/pamj.2017.26.70.11508

**Published:** 2017-02-20

**Authors:** Ababacar Abdoulaye Traore, Alami Badreeddine

**Affiliations:** 1 Service de Radiologie du CHU Hassan II, Fès, Maroc

**Keywords:** Volumineuse cholécystite aigue, dilatation kystique de la voie, Maroc, Voluminous acute cholecystitis, cystic dilatation of the common bile duct and imaging, Morocco

## Image en médecine

Il s’agit d’une patiente de 81 ans, admise au service des urgences pour prise en charge d’une colique hépatique associée à un ictère cutanéo-muqueux, quelques épisodes de vomissement et une hyperthermie. A l’examen physique, la patiente avait une sensibilité de l’hypochondre droit avec palpation d’une masse mobile douloureuse du flanc homolatéral arrivant jusqu’au niveau de la fosse iliaque droite. Un bilan biologique a montré une hyperleucocytose à polynucléaire neutrophile 12000/uL, une C Réactive Protéine à 144 mg/l avec perturbation du bilan hépatique de cholestase montre (élevation de la bilirubine et des transaminases à X fois la normale (pas besoin des chiffres exacts). Une échographie réalisée en urgence a montré une vésicule biliaire très distendue (14x07cm), lithiasiques à paroi épaissie, étendue en bas jusqu’au niveau du carrefour ilio caecal avec ectasie des voies biliaires (voie biliaire principale = 26mm). Une TDM abdominale a objectivé les mêmes résultats décrits à l’échographie, avec dilatation kystique du cholédoque, le conduit pancréatique principal était de calibre normal. Une CPRE réalisée avec sphinctorotomie a confirmé la dilatation kystique de la VBP. Le diagnostic de cholécystite aigue associé à une dilatation kystique de la VBP de type I de tadoni est retenu. Après antibiothérapie le patient a bénéficié d’un traitement chirurgical qui a consisté en une cholécystectomie avec exérèse en bloc de la voie biliaire dilatée, suivie d’une dérivation bilio-digestive sur anse en Y. Les suites opératoires étaient simples.

**Figure 1:**
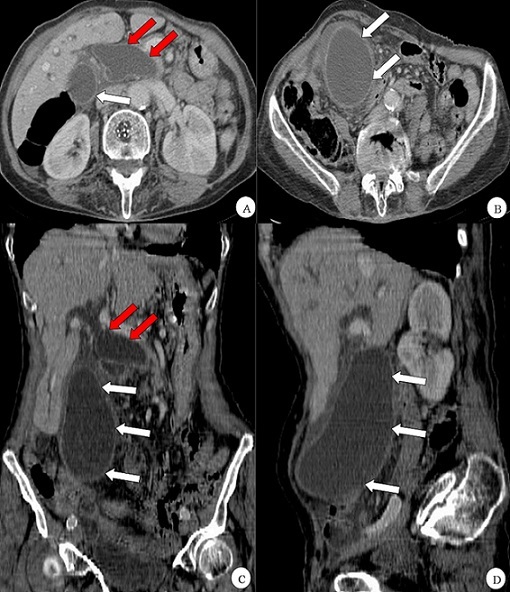
coupes TDM en coupes axiales (A, B), et reconstructions coronale (C) et sagittale (D) montre une volumineuse vésicule biliaire distendue, à paroi épaissie, arrivant en bas arrivant en bas jusqu’au niveau de la FID (flèche blanche) avec visualisation d’une dilatation kystique de la VBP (flèche rouge)

